# Abundant CD4+FOXP3+ regulatory T cells fail to suppress the proliferation of T cells in patients with Turner syndrome

**DOI:** 10.1186/1687-9856-2015-S1-O25

**Published:** 2015-04-28

**Authors:** Young Ah Lee, Hang-Rae Kim, Jeong Seon Lee, Haewoon Jung, Hwa Young Kim, Kyung Min Lee, Ji Hyun Sim, Doo Hyun Chung, Choong Ho Shin, Sei Won Yang

**Affiliations:** 1 Department of Pediatrics, Seoul National University Children's Hospital, Seoul, Korea; 2 Department of Anatomy, Seoul National University College of Medicine, Seoul, Korea; 3 Department of Biomedical Sciences, Seoul National University College of Medicine, Seoul, Korea; 4 Department of Pathology, Seoul National University Hospital, Seoul, Korea; 5 Ischemic/Hypoxia Institute, Seoul National University College of Medicine, Seoul, Korea

## Context

Why Turner syndrome (TS) patients are predisposed to autoimmune disease remains unclear.

## Objective

We investigated whether the frequency, phenotype, and suppressive function of CD4^+^FOXP3^+^ regulatory T cells (Tregs) are altered in young TS patients with the 45,X karyotype compared to age-matched controls.

## Methods

Peripheral blood mononuclear cells from young TS patients (n = 24, 17.4–35.9 yrs) and controls (n = 29) were stained with various Treg markers to characterize their phenotypes. Tregs sorted for CD4^+^CD25^bright^ were co-cultured with autologous CD4^+^CD25^−^ target cells in the presence of anti-CD3 and CD28 antibodies to assess their suppressive function.

## Results

TS patients exhibited a higher frequency of CD4^+^FOXP3^+^ Tregs among their lymphocytes (mean 2.06 vs. 1.52%, P = 0.005) and FOXP3^+^ Tregs among their CD4^+^ T cells (7.44 vs. 4.19%, P < 0.001) compared to controls. The expression of inhibitory CTLA-4 in the Tregs of TS patients was also significantly higher (mean fluorescence intensity = 214.1 vs. 184.6, P = 0.003). The frequency of Tregs expressing GITR^+^, CXCR3^+^, and CCR4^+^CCR6^+^ was comparable between the two groups. However, the ability of Tregs to suppress the in vitro proliferation of autologous CD4^+^CD25^−^ T cells was significantly impaired in TS patients compared to controls (P < 0.05 at a 0.1:1 ratio of Tregs to target cells, P < 0.01 at 0.25:1, 0.5:1, and 1:1).

## Conclusions

The Tregs of TS patients could not efficiently suppress the proliferation of autologous effector T cells, despite the abundance of Tregs in the peripheral circulation.

